# Secukinumab treatment in a pediatric patient with corticosteroid-intolerant pyoderma gangrenosum: a case report

**DOI:** 10.3389/fmed.2025.1676980

**Published:** 2025-10-02

**Authors:** Wang Liwei, Qian Yihong, Tan Fei, Yuan Chao

**Affiliations:** ^1^Skin & Cosmetic Research Department, Skin Disease Hospital of Tongji University, Shanghai, China; ^2^Skin Disease Hospital of Tongji University, Shanghai, China

**Keywords:** pyoderma gangrenosum, secukinumab, IL-17 inhibitor, pediatric, corticosteroid

## Abstract

Pyoderma gangrenosum (PG) is a rare, rapidly progressive, non-infectious neutrophilic dermatosis characterized by severely painful ulcers. Its pathogenesis may be attributed to genetic factors, autoimmune dysfunction, and neutrophil disorders. Interleukin-17 (IL-17) inhibitors can suppress neutrophil activity by blocking the IL-17/IL-23 axis, which has been documented to be effective in PG treatment. However, there is limited response rate of treatments with the use of IL-17 inhibitors for PG. Accordingly, the present study reports on a pediatric case of corticosteroid-intolerant pyoderma gangrenosum treated with the IL-17A inhibitor secukinumab, where the patient achieved significant improvement. This case underscores that secukinumab may be a promising therapeutic option for pediatric PG, offering valuable insights for clinical practice in managing such refractory cases.

## Introduction

Pyoderma gangrenosum (PG) is a rare, rapidly progressive, non-infectious neutrophilic dermatosis characterized by severely painful ulcers. The pathogenesis of this disease may involve genetic factors, autoimmune dysfunction, and neutrophil dysregulation. IL-17 inhibitors have been demonstrated to suppress the activity of neutrophils by blocking the IL-17/IL-23 axis. However, there is still suboptimal response rate in many case reports, and there is insufficient clinical experience with IL-17 inhibitors for PG. Herein, we reported a pediatric case of corticosteroid-intolerant PG treated with secukinumab. This report is anticipated to provide a novel therapeutic option for the management of pediatric PG.

## Clinical data

**Figure 1:**
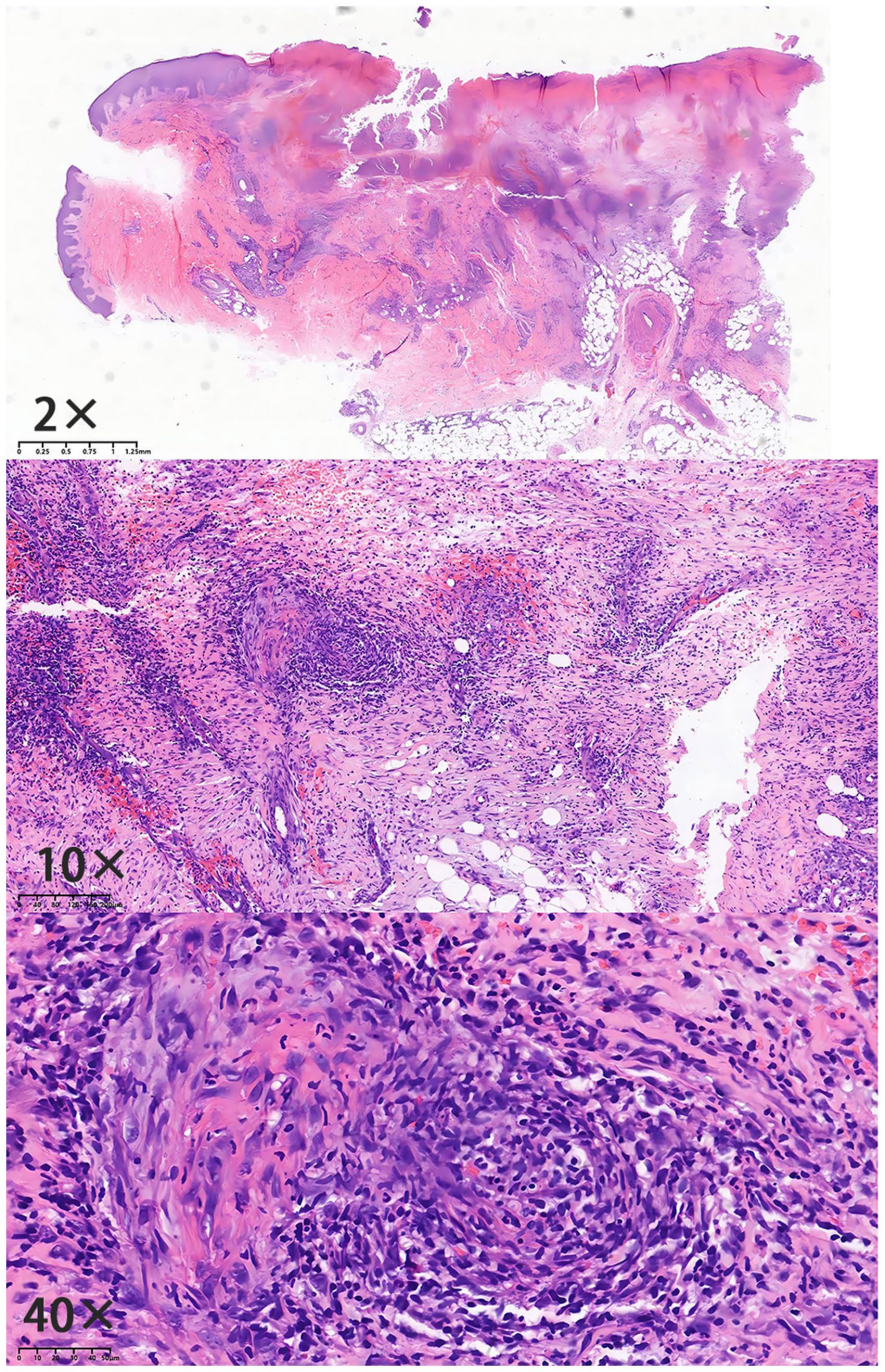
Histopathological findings (Specimen No.: D21-13321): epidermal ulceration with infiltration of neutrophils and formation of serocrust; edema of the upper dermal stroma; dense infiltration of lymphocytes, histiocytes, and neutrophils in the dermis and subcutaneous fat layer; indistinct vascular wall structure with erythrocyte extravasation; and stromal collagen hyperplasia with prominent red staining.

A male patient presented with recurrent erythema, papules, ulcers, and pruritus on both lower limbs since the age of 12, lasting for over 3 years, with worsening in the past 2 months. In January 2021, the patient developed dark red patches on both calves, with multiple ulcerated areas and exudate for no apparent reason. The patient was diagnosed with soft tissue infection in a local hospital initially, and treated with topical Mupirocin Ointment, which proved ineffective, with continuous expansion of these ulcers. In September 2021, the patient received a skin tissue biopsy of the lower extremity (Figure 1). Subsequently, the patient experienced recurrent condition intermittently. Throughout the course of the disease, the patient reported no history of abdominal pain, diarrhea, or joint pain. Furthermore, the patient had no preexisting systemic diseases, no specific details in his personal history, and no history of extensive or long-term oral medication use. His father had a history of photosensitivity.

The patient showed no abnormalities in systemic examination. Dermatological examination: Diffuse congestive erythema was observed on both lower extremities, with swelling over the dorsum of the feet, desquamation at the foot margins, and densely distributed millet-sized papules and papulovesicles. Several ulcers with irregular borders and partially covered by purulent exudate were noted on the extensor aspect of the left lower leg and heel, surrounded by a violaceous-red halo, with positive tenderness and slightly elevated skin temperature. The right lower extremity was covered with old scars and pigmentation. Meanwhile, there was no abnormality in the mucosa of the external genitalia or oral cavity; or deformity and tenderness in the joints of the limbs.

To further rule out infectious ulcers, vascular ulcers, and autoimmune ulcers, we completed the following examinations and tests during the patient’s consultation. Initial tests for fungal culture, bacterial culture, total IgE, and ANCA were all negative. Based on the integration of the patient’s medical history, clinical symptoms, physical signs, and pathological findings, the diagnosis was confirmed as pyoderma gangrenosum (PG).

**Figure 2:**
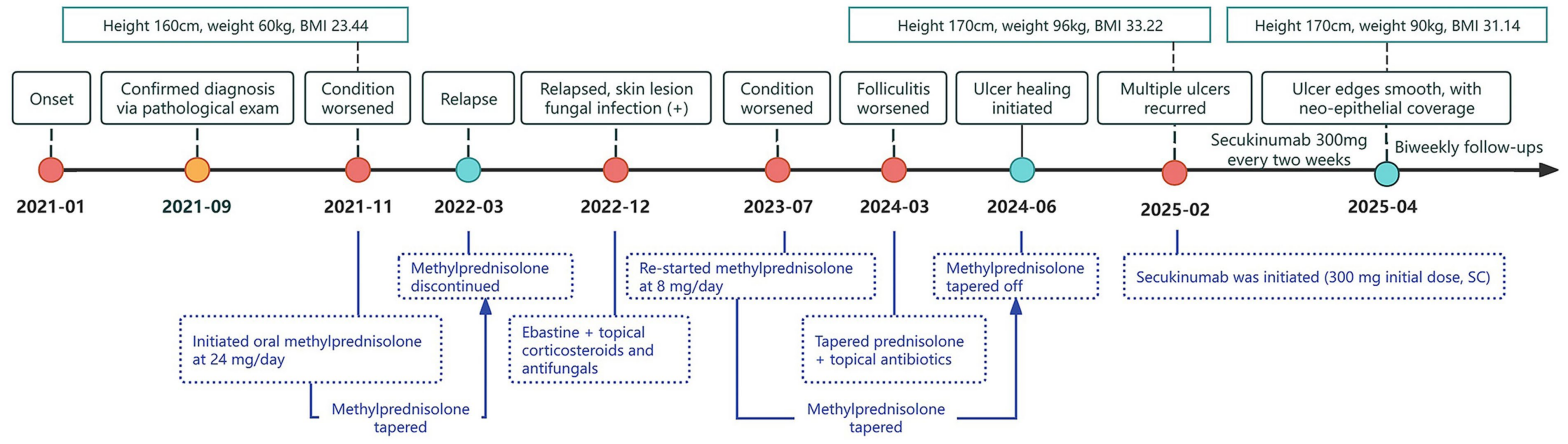
Timeline of treatment milestones during 2021–2025. The timeline illustrates the pediatric patient’s weight fluctuations and key treatment milestones from January 2021 to April 2025. The patient’s height, weight, and BMI (kg/m^2^) are indicated above the timeline, while key events such as disease progression and treatment adjustments are represented by color-coded dots and arrows below. Yellow dots denote confirmation of diagnosis; red dots denote disease exacerbation or recurrence; green dots denote disease remission.

During the disease course, the patient initiated oral methylprednisolone in November 2021, with initial symptom improvement. However, the skin lesions recurred repeatedly with each glucocorticoid taper. Due to being in a phase of active growth and development, the patient failed to achieve complete remission of the skin lesions after glucocorticoid maintenance therapy and concurrently experienced a rapid increase in body weight. Moreover, the patient experienced recurrent fungal and bacterial skin infections during the treatment course (Figure 2).

**Figure 3:**
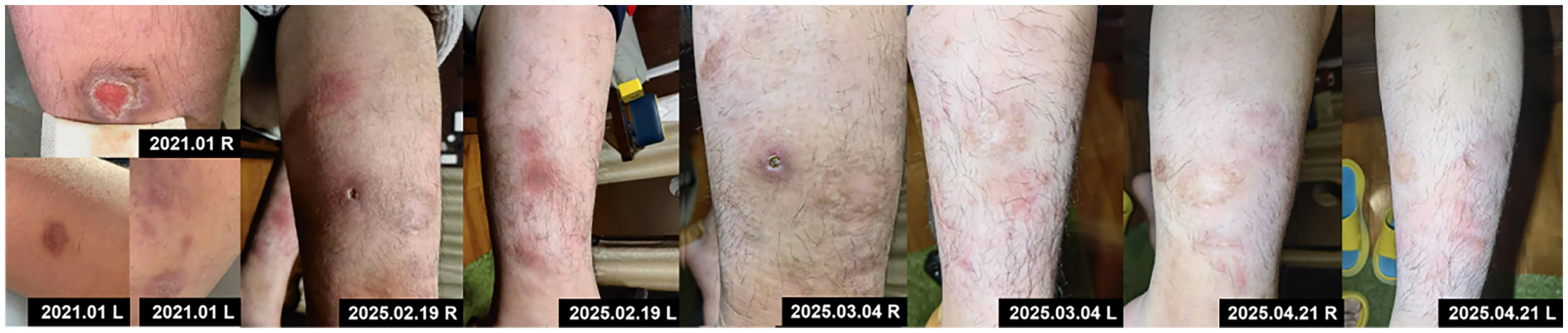
Initial skin lesions and changes following secukinumab treatment at five treatment timepoints of 2025.

On February 15, 2025, after obtaining informed consent from both the patient and guardians and the exclusion of contraindications, the patient was given treatment with secukinumab, an IL-17A inhibitor, given the relapse and worsening of skin lesions. The initial dose was 150 mg × 2 (300 mg) through subcutaneous injection, followed by 300 mg once weekly for maintenance, and was concomitantly treated with topical antibiotics. After 2 weeks of secukinumab treatment, the patient showed significantly subsided erythema and edema in both lower extremities, decreased ulcer exudation, and relieved pruritus. By week 4, the patient was observed with smoother ulcer margins, and re-epithelialization in the central areas, forming scar tissues. By week 8, the patient experienced ulcer healing, with residual erythema present at the sites of prior lesions (Figure 3). During treatment, routine CBC as well as hepatic and renal function monitoring revealed no abnormalities in the patient, without any adverse events such as infections or allergic reactions. At follow-up in May 2025, the patient was in stable condition, and was prescribed secukinumab injections every 4 weeks for maintenance treatment, with regular follow-up advised.

## Discussion

PG is a rare neutrophilic dermatosis, which is estimated to affect 5.8 cases per 100,000 adults (1, 2). PG significantly impairs patients’ quality of life and is associated with a threefold increased risk of mortality compared to the general population (3). PG consists of four subtypes (e.g., ulcerative, vegetative, bullous, and pustular), with the ulcerative form being the most common. Skin lesions typically begin as papules, pustules, or nodules, which rapidly ulcerate and evolve into progressively enlarging, non-infectious deep ulcers, with red to violaceous undermined borders and severe pain (4). The lower extremities are the major sites of occurrence, involving approximately 80% of cases (5). The diagnosis is primarily made by excluding other causes of skin ulcers, coupled with clinical presentation and histopathological features for further confirmation. Despite an unclear understanding of the precise pathogenesis, PG is thought to be multifactorial, involving immune dysregulation, genetic predisposition, infections, and associated systemic diseases. About 50% of PG patients have comorbidities such as inflammatory bowel disease or rheumatoid arthritis (6). In this case, the patient presented with erythema and painful ulcers on both lower limbs. Further findings of ulcerative lesions with predominant neutrophilic infiltration, indicated by histopathological examination, were well consistent with the diagnostic criteria for ulcerative-type PG. Besides, the patient was identified without other systemic comorbidities.

According to recent research, skin lesions in PG exhibited remarkably upregulated levels of inflammatory cytokines, including IL-8, IL-17A, IL-23A, TNF-α, IFN-γ, and CXCL-1/2/3/9/10/11/16 (7), accompanied by an increased Th17/Treg cell ratio. Elevated serum levels of IL-6, IL-8, IL-17, and IL-23 were frequently reported in PG patients as well (8–10). Mechanistically, activation of the Th17 cell axis, particularly the IL-17/IL-23 pathway, is pivotal in triggering the inflammatory cascade. IL-23 secreted by dendritic cells can promote Th17 cell expansion and IL-17A production via the JAK 2–STAT3 signaling pathway, leading to massive neutrophil recruitment and activation, thereby driving tissue damage (11).

Currently, no standardized therapeutic protocol is available for the management of PG. Traditional therapies primarily involve the use of systemic glucocorticoids and immunosuppressive agents. In recent decades, small-molecule drugs and biologic agents have emerged as promising alternatives, showing encouraging therapeutic outcomes in case reports and small studies (4). JAK inhibitors, such as tofacitinib and ruxolitinib, have demonstrated efficacy in PG treatment. The phosphodiesterase-4 inhibitor apremilast also shows potential as a monotherapy for refractory PG and may be used in combination with other agents (12, 13). Meanwhile, as for specific biologic agents, TNF-α inhibitors (e.g., infliximab and adalimumab), are effective options and may serve as alternatives to long-term use of corticosteroids or cyclosporine to mitigate relevant adverse effects (14, 15). Simultaneously, IL-1 inhibitors also exhibit therapeutic potential for PG in case reports and open-label studies (16). Secukinumab, brodalumab and other IL-17 inhibitors might also exert potential efficacy in some patients with PG (17); however, there were also reports of PG being paradoxically induced by this drug class, particularly in switch-therapy settings or certain predisposing conditions (18). Additionally, IL-23 inhibitors, such as ustekinumab and guselkumab, have demonstrated a therapeutic effect in the majority of patients across several studies (19, 20).

In our case, an adolescent patient with PG showed poor response to conventional therapy, with persistent, non-healing skin lesions that repeatedly worsened over time. During his growth and development period, the physical development of this patient was compromised by long-term maintenance with oral glucocorticoids, leading to rapid weight gain, coupled with recurrent bacterial and fungal infections. Noticeably, the safety profile of the therapeutic agent was a critical concern given the patient’s age and history of recurrent infections. Accordingly, this patient was provided with secukinumab, an agent approved for moderate-to-severe plaque psoriasis in pediatric ≥6-year-old patients, with supportive safety data in children. For instance, in a phase I–II pilot study, seven patients received secukinumab 300 mg weekly from week 0 to 4, followed by 300 mg every 4 weeks in two patients through week 16, and every 2 weeks in five patients through week 32. Notably, two patients continued treatment through week 32 and experienced significant improvement in ulcer size, inflammatory markers, and Dermatology Life Quality Index scores; all participants reported pain relief (17). Following this dosing regimen, our patient exhibited marked improvement in skin lesions, weight reduction, and pain alleviation. Furthermore, our literature review identified six case reports on the use of secukinumab for PG treatment (21–26), all of which reported clinical improvement, though all involved adult patients.

This case report demonstrates that secukinumab, an IL-17A inhibitor, achieved ulcer healing and symptom relief without adverse reactions in an adolescent with corticosteroid-intolerant PG over an 8-week short-term treatment course, providing practical single-case reference for managing refractory pediatric PG. However, as a single case report, it has inherent limitations: it cannot extrapolate secukinumab’s universal efficacy in pediatric PG from one patient’s short-term data, nor fully address the potential paradoxical reactions or long-term adverse events associated with IL-17 inhibitors. With follow-up limited to 3 months (through May 2025), short-term safety findings cannot rule out delayed risks such as increased infection susceptibility. Furthermore, the long-term impact of IL-17 pathway blockade on pediatric growth and immune maturation remains unclear, and secukinumab’s established safety data in children with psoriasis cannot be directly generalized to PG. Consequently, this case offers preliminary clinical clues but no definitive evidence for secukinumab’s efficacy or long-term safety in pediatric PG; multi-center, long-term studies are therefore necessary to validate the role of IL-17 inhibitors in this patient population.

## Data Availability

The raw data supporting the conclusions of this article will be made available by the authors, without undue reservation.

## References

[1] Xu A Balgobind A Strunk A Garg A Alloo A. Prevalence estimates for pyoderma gangrenosum in the United States: an age- and sex-adjusted population analysis. J Am Acad Dermatol. (2020) 83:425–9. doi: 10.1016/j.jaad.2019.08.001, PMID: 31400451

[2] Orfaly VE Reese AM Friedman M Latour E Ortega-Loayza AG. Pyoderma gangrenosum study pilot registry: the first step to a better understanding. Wound Repair Regen. (2022) 30:334–7. doi: 10.1111/wrr.13005, PMID: 35363927

[3] Langan SM Groves RW Card TR Gulliford MC. Incidence, mortality, and disease associations of pyoderma gangrenosum in the United Kingdom: a retrospective cohort study. J Invest Dermatol. (2012) 132:2166–70. doi: 10.1038/jid.2012.130, PMID: 22534879

[4] Zaino ML Schadt CR Callen JP Owen LG. Pyoderma gangrenosum: diagnostic criteria, subtypes, systemic associations, and workup. Dermatol Clin. (2024) 42:157–70. doi: 10.1016/j.det.2023.08.003, PMID: 38423678

[5] Binus AM Qureshi AA Li VW Winterfield LS. Pyoderma gangrenosum: a retrospective review of patient characteristics, comorbidities and therapy in 103 patients. Br J Dermatol. (2011) 165:1244–50. doi: 10.1111/j.1365-2133.2011.10565.x, PMID: 21824126

[6] Yamamoto T. Epidemiology of pyoderma gangrenosum in Japanese patients by questionnaire survey. J Dermatol. (2019) 46:e145–6. doi: 10.1111/1346-8138.14658, PMID: 30230578

[7] Marzano AV Damiani G Ceccherini I Berti E Gattorno M Cugno M. Autoinflammation in pyoderma gangrenosum and its syndromic form (pyoderma gangrenosum, acne and suppurative hidradenitis). Br J Dermatol. (2017) 176:1588–98. doi: 10.1111/bjd.15226, PMID: 27943240

[8] Ortega-Loayza AG Friedman MA Reese AM Liu Y Greiling TM Cassidy PB. Molecular and cellular characterization of pyoderma gangrenosum: implications for the use of gene expression. J Invest Dermatol. (2022) 142:1217–1220.e14. doi: 10.1016/j.jid.2021.08.431, PMID: 34536481 PMC12923208

[9] Guenova E Teske A Fehrenbacher B Hoerber S Adamczyk A Schaller M. Interleukin 23 expression in pyoderma gangrenosum and targeted therapy with ustekinumab. Arch Dermatol. (2011) 147:1203–5. doi: 10.1001/archdermatol.2011.168, PMID: 21680759

[10] Rubas K Reich A Nowicka-Suszko D Maj J. The role of interleukins 6, 8, 17 and 23 in the pathogenesis of pyoderma gangrenosum. J Eur Acad Dermatol Venereol. (2023) 37:e660–2. doi: 10.1111/jdv.18683, PMID: 36268700

[11] Maverakis E Marzano AV Le ST Callen JP Brüggen MC Guenova E. Pyoderma gangrenosum. Nat Rev Dis Primers. (2020) 6:81. doi: 10.1038/s41572-020-0213-x, PMID: 33033263

[12] Bordeaux ZA Kwatra SG West CE. Treatment of pyoderma gangrenosum with apremilast monotherapy. JAAD Case Rep. (2022) 30:8–10. doi: 10.1016/j.jdcr.2022.10.001, PMID: 36345410 PMC9636013

[13] Laird ME Tong LX Sicco KIL Kim RH Meehan SA Franks AG. Novel use of apremilast for adjunctive treatment of recalcitrant pyoderma gangrenosum. JAAD Case Rep. (2017) 3:228–9. doi: 10.1016/j.jdcr.2017.02.019 28443317 PMC5394202

[14] Ben AH Fogh K Bech R. Pyoderma gangrenosum and tumour necrosis factor alpha inhibitors: a semi-systematic review. Int Wound J. (2019) 16:511–21. doi: 10.1111/iwj.13067 30604927 PMC7949186

[15] Yamamoto T. An update on adalimumab for pyoderma gangrenosum. Drugs Today (Barc). (2021) 57:535–42. doi: 10.1358/dot.2021.57.9.3293619, PMID: 34586101

[16] McKenzie F Cash D Gupta A Cummings LW Ortega-Loayza AG. Biologic and small-molecule medications in the management of pyoderma gangrenosum. J Dermatolog Treat. (2019) 30:264–76. doi: 10.1080/09546634.2018.1506083, PMID: 30051737

[17] Lauffer FSPBD. 044 safety and efficacy of anti-IL-17 (secukinumab) for the treatment of pyoderma gangrenosum. J Invest Dermatol. (2021) 141:S156. doi: 10.1016/j.jid.2021.08.046, PMID: 40963457

[18] Sadik CD Thieme M Zillikens D Terheyden P. First emergence of pyoderma gangraenosum, palmoplantar pustulosis and sacroiliitis in a psoriasis patient associated with switching from secukinumab to brodalumab. J Eur Acad Dermatol Venereol. (2019) 33:e406–7. doi: 10.1111/jdv.15714, PMID: 31131924

[19] Westerdahl JS Nusbaum KB Chung CG Kaffenberger BH Ortega-Loayza AG. Ustekinumab as adjuvant treatment for all pyoderma gangrenosum subtypes. J Dermatolog Treat. (2022) 33:2386–90. doi: 10.1080/09546634.2021.1937475, PMID: 34057010

[20] Ben Abdallah H Fogh K Vestergaard C Bech R. Pyoderma gangrenosum and interleukin inhibitors: a semi-systematic review. Dermatology. (2022) 238:785–92. doi: 10.1159/000519320 34710873

[21] Orita A Hoshina D Hirosaki K. Pyoderma gangrenosum caused by secukinumab successfully treated with risankizumab: a case report and literature review. Clin Exp Dermatol. (2022) 47:1372–4. doi: 10.1111/ced.15183, PMID: 35298047

[22] Coe J Kudva S Shams K. Matching the dose to the disease: successful treatment of recalcitrant pyoderma gangrenosum using high-dose secukinumab. Dermatol Ther. (2022) 35:e15669. doi: 10.1111/dth.15669, PMID: 35762275

[23] Li M Xiang H Liang Y Xue L Zhou X Yang L. Secukinumab for PASS syndrome: a new choice for therapeutic challenge? Dermatol Ther. (2022) 35:e15507. doi: 10.1111/dth.15507, PMID: 35419914

[24] Maronese CA Pimentel MA Li MM Genovese G Ortega-Loayza AG Marzano AV. Pyoderma gangrenosum: an updated literature review on established and emerging pharmacological treatments. Am J Clin Dermatol. (2022) 23:615–34. doi: 10.1007/s40257-022-00699-8, PMID: 35606650 PMC9464730

[25] McPhie ML Kirchhof MG. Pyoderma gangrenosum treated with secukinumab: a case report. SAGE Open Med Case Rep. (2020) 8:2050313X-20940430X. doi: 10.1177/2050313X20940430, PMID: 32733679 PMC7370553

[26] Nikolakis G Kreibich K Vaiopoulos A Kaleta K Talas J Becker M. Case report: PsAPSASH syndrome: an alternative phenotype of syndromic hidradenitis suppurativa treated with the IL-17A inhibitor secukinumab. F1000Res. (2021) 10:381. doi: 10.12688/f1000research.52100.2, PMID: 34540202 PMC8424462

